# Synthesis of Marine (−)-Pelorol and Future Perspectives

**DOI:** 10.3390/md22090425

**Published:** 2024-09-19

**Authors:** Antonio Rosales Martínez, Ignacio Rodríguez-García

**Affiliations:** 1 Department of Chemical Engineering, Escuela Politécnica Superior, University of Sevilla, 41011 Sevilla, Spain; 2 Organic Chemistry, CeiA3, CIAIMBITAL, University of Almería, 04120 Almeria, Spain; irodrigu@ual.es

**Keywords:** marine natural products, meroterpenoids, pelorol

## Abstract

Meroterpenoid-type marine natural compounds have attracted an increasing amount of attention due to their peculiar chemical structures and their potential for the development of therapeutically important probes. Within this group of substances pelorol stands out; it is a natural compound isolated from marine organisms with a unique structure and an interesting biological profile. In this article, we summarize and highlight the most interesting aspects of the synthetic procedures towards this compound, which have two common key steps. The first is the coupling of a drimanyl derivative with a compound derived from an arene. The second is a Friedel–Crafts cyclization which forms the C ring of the natural product. Despite the synthetic advances achieved so far, we consider that a more efficient synthetic procedures could be carried out, since their synthetic routes are difficult to scale up due to numerous reaction steps and the limitations imposed by the use of some reagents. In this article, we present a new and versatile retrosynthetic analysis of (−)-pelorol and analogs, which is highly desirable for their easy preparation and subsequent broad study of their biological activities. This is a retrosynthetic route that could improve those reported in the literature in terms of cost-effectiveness.

## 1. Introduction

**Figure 1:**
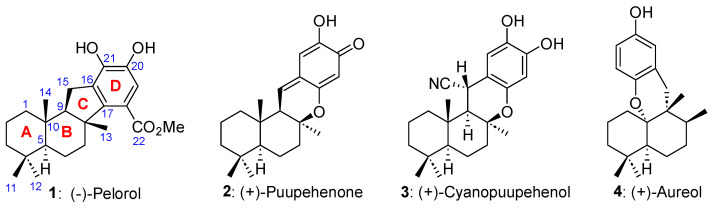
Representative bioactive drimane meroterpenoids.

Drimane meroterpenoids are defined as compounds of mixed terpenoid–polyketide origin [1,2,3] (Figure 1). These natural compounds have been isolated from marine organisms, particularly from algae and sponges [4,5,6], and they are excellent examples of natural products with structural diversity having interesting biological activities, such as anti-HIV [7], antibacterial [8], antitumor [9,10,11], antifungal [12], etc. [13,14]. For these reasons, these fascinating compounds have attracted widespread attention from synthetic chemical pharmacologists and biologists. It should be noted that the efforts made in natural products synthesis generates synergistic effects with synthetic methodology, since the results obtained help the general development of chemical synthesis.

**Scheme 1:**
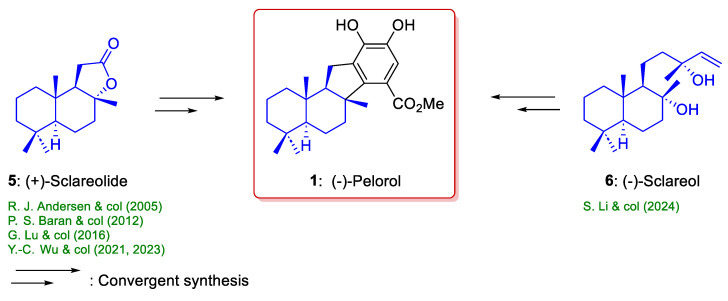
Chiral starting materials used in previous syntheses of (−)-pelorol (**1**): (+)-sclareolide [19,21,24,25,26] and (−)-sclareol [20].

Within the marine meroterpenoid family, (−)-pelorol (**1**) is a remarkable compound which integrates a sesquiterpenoid unit and a phenolic moiety. This natural marine compound, which was initially isolated from *Dactylospongia elegants* [15] and later from *Petrosaspongia metachromia* [16] and *Hyrtros erectus* [17], is bioactive against *Plasmodium* and *trypanosome*, showing significant activity against *P. falciparum* with an IC50 value of 0.8 μM [15,16], has insecticidal activity [16,17], exhibits cytotoxicity against HeLa cancer cells [18], has anti-inflammatory activity by activating inositol-5-phosphatase (SHIP) [19], and possesses good antifungal activity against *Rhizoctonia solani*, with EC50 values of 7.7 μM [20]. Of the various biological activities mentioned, the anti-cancer activity deserves special mention. Previous studies demonstrated that (−)-pelorol (**1**) inhibits the enzyme phosphatidylinositol 3-kinase (PI3) [21] and has anti-inflammatory activity because it can activate inositol-5-phosphatase (SHIP) [19]. Due to the fact that the deactivation of aberrant PI3K signaling in cancer cells, by the activation of SHIP, has been proposed to be a promising approach to treating blood cancers [22,23] (−)-pelorol (**1**) can be considered as a promoting lead compound for therapeutic development. Several elegant syntheses of (−)-pelorol (**1**) have employed a convergent synthetic route (Scheme 1), where two fragments are linked to form the desired marine compound, normally by the addition of an aryl-metallic reagent to a sesquiterpenoid carrying a carbonyl group through a Suzuki coupling or a palladium-catalyzed carbene migratory insertion (see Section 2). In all cases, the starting materials are commercially available precursors, such as (+)-sclareolide (**5**) or (−)-sclareol (**6**). These starting materials selected for the preparation of **1** exhibit the same absolute configurations at C-5, C-9, and C-10 as those predicted for (−)-pelorol (**1**). Scheme 1 summarizes the chiral starting material selected in the synthesis of **1** by convergent synthetic routes [22].

This article highlights the previous approaches to (−)-pelorol (**1**), and finally, a strategy to access to this natural product from a common intermediate that can be employed in the total divergent synthesis of natural products will be discussed.

## 2. Synthesis of (−)-Pelorol (1)

### 2.1. Andersen’s Synthesis of (−)-Pelorol (***1***)

**Scheme 2:**
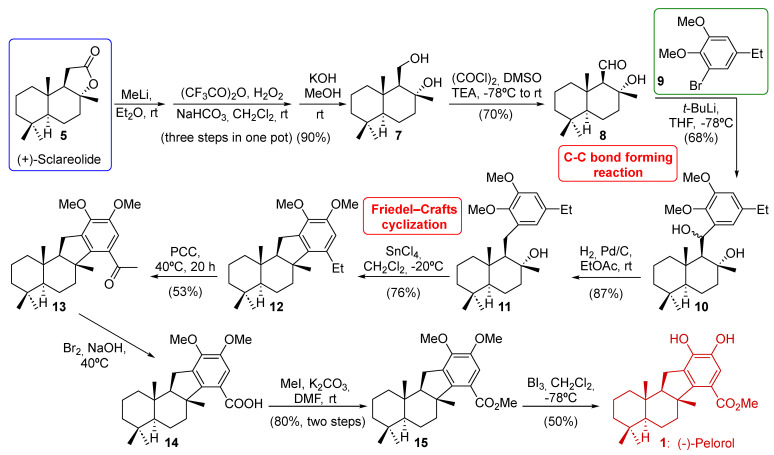
Andersen’s enantioselective synthesis of (−)-pelorol (**1**) from (+)-sclareolide (**5**). DMSO = dimethyl sulfoxide; PCC = pyridinium chlorochromate; DMF = *N*,*N*-dimethyl formamide.

In 2005, Andersen’s group [19] reported the first enantioselective synthesis of natural (−)-pelorol (**1**) via an addition of an aryl-metallic reagent formed from **9** to the carbonyl compound **8** as a key step (Scheme 2). Andersen’s synthesis of **1** used (+)-sclareolide (**5**) as the starting material. The diol **7** was synthesized in three steps in one pot from (+)-sclareolide (**5**) through a degradative sequence of reactions (90% overall yields). Its oxidation under Swern conditions gave the β-hydroxy drimanaldehyde **8** in 70% yield. Then, the coupling reaction between **8** and the organolithium derived from **9** gave, after hydrogenolysis, the tertiary alcohol **11** as key compound. The Friedel–Crafts-type cyclization of **11** with SnCl_4_ afforded the indane-fused decalin **12** as cyclization product in 76% yield. The oxidation of compound **12** with PCC selectively oxidized the C-22 methylene to form methyl ketone **13** in a moderate yield. The treatment of **13** with Br_2_ gave the desired benzoic acid **14**, which after esterification with MeI gave the compound **15** with an 80% yield (two steps). Finally, the selective cleavage of the phenyl methyl ethers with BI_3_ at −78 °C completed the synthesis of (−)-pelorol (**1**) at a 50% yield. This total synthesis was achieved in 11 steps (6% overall yield) from starting material **5**. The strategy opens the door to the preparation of an array of different natural products through a modular strategy, through the reaction of **8** with a choice of aryl bromides similar to **9**. The major drawbacks of this approach are the moderate yield of the degradative oxidation of **12** and the need to use of protective groups for the phenolic hydroxy groups. Also, this synthetic route is difficult to scale up due to the numerous steps.

### 2.2. Baran’s Synthesis of (−)-Pelorol (***1***)

**Scheme 3:**
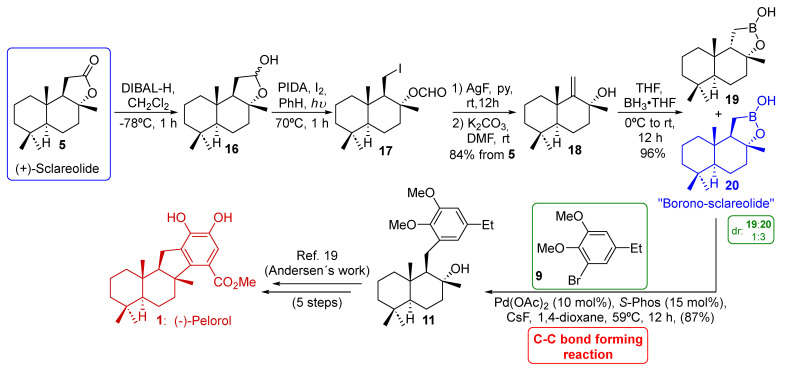
Baran’s enantioselective synthesis of (−)-pelorol (**1**) from (+)-sclareolide (**5**). DIBAL-H = diisobutylaluminium hydride; PIDA = phenyliodine (III) diacetate; DMF = *N*,*N*-dimethyl formamide.

Baran and colleagues [24] reported the formal synthesis of the (−)-enantiomer of pelorol (**1**) from (+)-sclareolide (**5**) as a starting material. Their approach was based on the synthesis of a “borono-sclareolide” **20** as a common pluripotent intermediate widely used in the divergent synthesis of meroterpenoids. As shown in Scheme 3, the key intermediate **20** required the excision of carbon monoxide from (+)-sclareolide (**5**) and the introduction of B-OH in its place. The reduction in starting material **5** using diisobutylaluminium hydride (DIBAL-H) gave sclareal **16**. The treatment of **16** under the C-C bond cleavage conditions of Suarez [27] delivered drimanal iodoformate **17**. Then, a two-step dehydroiodination/hydrolysis procedure (AgF in pyridine followed by K_2_CO_3_ in MeOH) gave the natural exocyclic decalin **18** with an excellent 84% overall yield from **5**. Compound **18** was synthesized on the decagram scale and was used without the need for technical chromatographic separation. The hydroboration of **18** with BH_3_ gave **19** and **20** as a mixture of diastereomers (**19**:**20** = 1:3) at a 96% yield. Borono-sclareolide **20** was synthesized at the gram-scale and purified by column chromatography. This compound is a versatile terpene donor, which can be used as a partner of aryl bromides in Suzuki coupling reactions for the preparation of meroterpenoids. The C-C bond forming reaction between **20** and aryl bromide **9** gave the tertiary alcohol **11** at a 87% yield. As alcohol **11** is an advanced intermediate in Andersen’s synthesis of (−)-pelorol (**1**) (Scheme 2) [19], this procedure constitutes a formal synthesis of **1**. In this way, the synthesis of **1** can be achieved in 11 steps (8.5% overall yield) from commercially available (+)-sclareolide (**5**).

The key component of this procedure was the invention of “borono-sclareolide” **20** as a common intermediate employed in the design of the divergent, modular, and scalable access of meroterpenoids and analogues [24]. The synthesis of the intermediate **20** represents an excellent example of how synthetic methodology can generate synergistic effects with natural product synthesis, since an efficient and versatile reaction development can also be of use in the preparation of natural products. One of the main limitations of this synthetic route is the use of a palladium catalyst which could be expensive.

### 2.3. Lu’s Synthesis of (−)-Pelorol (***1***)

**Scheme 4:**
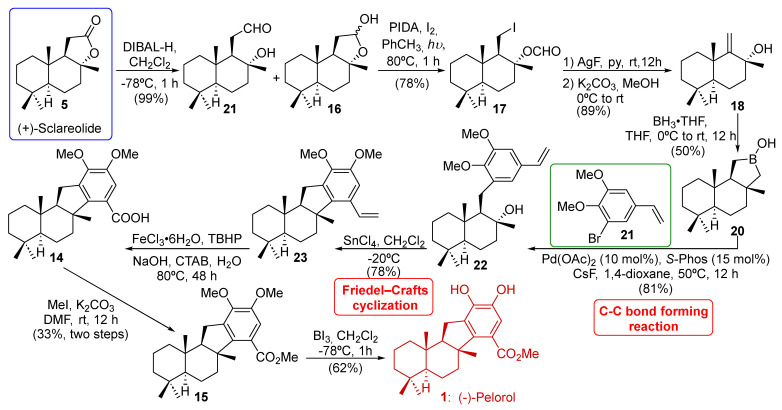
Lu’s enantioselective synthesis of (−)-pelorol (**1**) from (+)-sclareolide (**5**). DIBAL-H = diisobutylaluminium hydride; PIDA = phenyliodine (III) diacetate; TBHP = *tert*-Butyl hydroperoxide; CTAB = Cetrimonium bromide; DMF = *N*,*N*-dimethyl formamide.

In 2016, Lu and coworkers [21] accomplished another formal synthesis of (−)-pelorol (**1**) using the procedure previously developed by Baran et at. [24], but choosing a more efficient aryl bromide **21** as coupling partner of “bonoro-sclareolide” **20** (Scheme 4). The synthesis of **1** started with the reduction in available (+)-sclareolide (**5**) with DIBAL-H yielding a mixture of **21** and **16** with an excellent yield of 99%, which was used without separation in the next step. The iodination of the mixture under Suarez’s conditions [27], but replacing benzene with toluene, resulted in a 78% yield of **17**. A sequential dehydroiodination/hydrolysis reaction led to the formation of compound **18** at a 89% yield. Then, hydroboration of **18** gave a mixture of diastereomeric boronates (95% combined yield). Column chromatography allowed the isolation of pure borono-sclareolide **20** at a 50% yield. In a first instance, this research group used arylbromide with ethyl substituent (**9**) to form the C-C connection with the borono-sclareolide **20** under Suzuki conditions, but the oxidation of the intermediate **12**, obtained after the cyclization of the coupling product **11**, gave a poor yield (27%). Considering this inconvenience, this research group changed the arylbromide **9** to the vinyl-substituted arylbromide **21**, yielding the intermediate **22** (81%) under Suzuki coupling conditions. The subsequent Friedel–Crafts cyclization of **22** afforded the indane-fused decalin **23** with a good yield (78%). The oxidation of the vinyl group in **23** using a *tert*-butyl hydroperoxide (TBHP)-based procedure gave the acid **14**, which after esterification with MeI, generated the compound **15**, although at a low yield of 33% (two steps). The deprotection of the OMe group in **15**, with the procedure previously reported in Andersen’s synthesis [19], gave the desirable (−)-pelorol (**1**) at a 62% yield. As product **14** was an advanced intermediate in the Andersen’s synthesis of (−)-pelorol [19], this procedure described by Lu and coworkers constitutes a formal synthesis of the natural marine product **1**. This formal synthesis was completed in nine steps (4.4% overall yield) from commercially available (+)-sclareolide (**5**).

Again, in this synthetic route, the power of “borono-sclareolide” **20** for a divergent and scalable access to (−)-pelorol **1** was demonstrated. Furthermore, a better choice of aryl bromide **21** as coupling partner of **20** resulted in a more effective synthetic route.

### 2.4. Wu’s Synthesis of (−)-Pelorol (***1***)

**Scheme 5:**
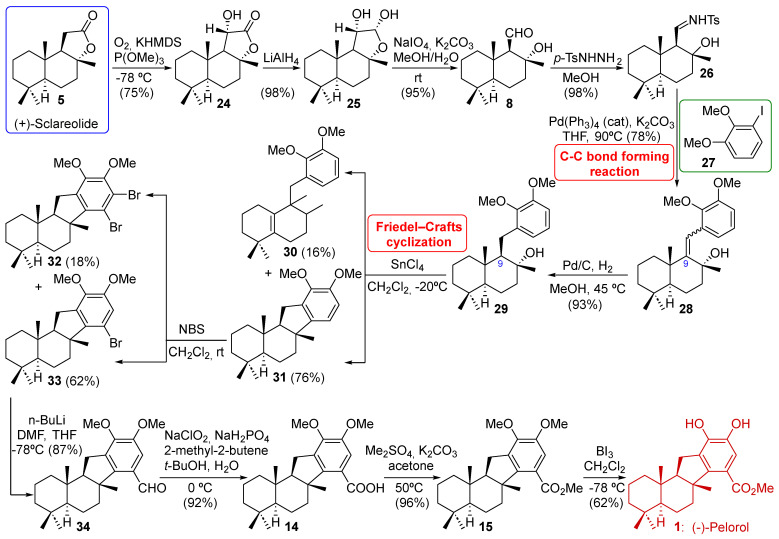
Wu’s first enantioselective synthesis of (−)-pelorol (**1**) from (+)-sclareolide (**5**). KHDMS = Potassium bis (trimethylsilyl)amide; NBS = *N*-Bromosuccinimide; DMF = *N*,*N*-dimethyl formamide.

In 2021, Wu’s group [25] achieved the formal synthesis of (−)-pelorol (**1**) through the development of a modular strategy for the synthesis of meroterpenoid marine compounds, having a palladium-catalyzed coupling of an aryliodide and a tosylhydrazone as key step [28]. As shown in Scheme 5, the key intermediate drimanal hydrazone **26** was prepared from (+)-sclareolide (**5**) in four steps (68% overall yield). The sequence includes the selective oxidation of **5** with O_2_ to give **24**, a reduction in **24** with LiAlH_4_ to form **25**, the subsequent cleavage of the diol **25** with NaIO_4_ to generate **8**, and finally, the formation of drimanal hydrazone **26** with *p*-TsNHNH_2_. A Pd(0)-catalyzed tandem carbene migratory insertion reaction allowed the cross-coupling of **26** and *ortho*-iodoveratrol (**27**), previously obtained from commercially available veratrole. Product **28** was formed as a mixture of isomers ((*Z*)-**28a** and (*E*)-**28b** in 37% and 41% yield, respectively). This methodology can be applied to the preparation of other meroterpenoids by simply choosing a different aryl iodide. The advantages of these couplings between hydrazone **26** and aryl iodides with different substitution patterns are that the reaction conditions are mild and that both partners can be easily synthesized, although the use of a palladium catalyst in this coupling limits the scale-up of synthetic strategy due to its high costs.

The next logical step should be cyclization to form ring C. However, to avoid the inversion of configuration at C-9 during the process, the double bond in **28** had to be hydrogenated prior to the cyclization. The reverse procedure (cyclization and subsequent hydrogenation) led to products with the opposite configuration at the C-9 position, and was used by Bisai and coworkers for the synthesis of 9-*epi*-pelorol [29]. The hydrogenation of the intermediates **28** gave **29** at a 93% yield. The Friedel–Crafts cyclization of **29** in the presence of SnCl_4_ allowed the formation of the desired C ring in the tetracyclic compound **31** in 76% yield, although a rearranged minor compound **30** was also formed with a 16% yield. The bromination of **31** with NBS gave monobrominated **33** as the major product (62% yield), together with the dibromoderivative **32** as a minor one (18% yield). The formylation of the bromo compound **33** was achieved with DMF in the presence of *n*-BuLi, which led to the formation of aldehyde **34** at an 87% yield. The Pinnick oxidation [29] of **34** yielded carboxylic acid **14** at a 92% yield, which was then methylated to form the ester **15** in 96% yield. Finally, the selective demethylation of **15**, using the conditions previously reported by Andersen in the synthesis of **1** [19], resulted in the marine (−)-pelorol (**1**) at a 62% yield. The formal synthesis of **1** was completed in 12 steps (9% overall yield) from available starting material (+)-sclareolide (**5**).

**Scheme 6:**
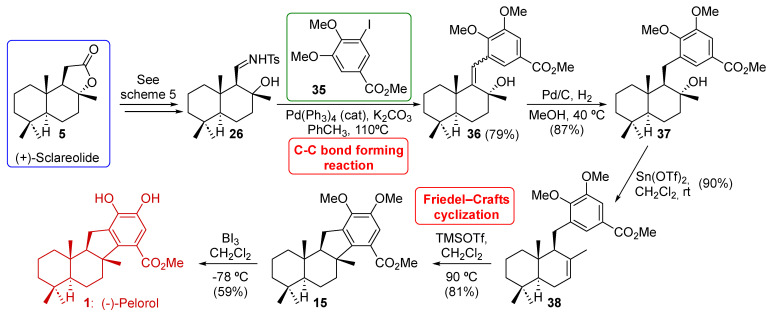
Wu’s second enantioselective synthesis of (−)-pelorol (**1**) from (+)-sclareolide (**5**).

Two years later, the same research group published [26] an improved synthesis of **1** using the same synthetic strategy but varying the aryliodide partner (Scheme 6). In the synthesis discussed above, the methoxycarbonyl group was introduced into the aromatic ring after creating the cyclopentane ring C, since the deactivating effect of the ester group would make rather difficult the Friedel–Crafts cyclization. Wu and colleagues [26] found that TMSOTf is an extremely powerful additive, which can efficiently promote the Friedel–Crafts process even in when the aryl ring has electron-withdrawing groups, such as an ester. For that reason, they chose the aryliodide **35** as cross-coupling partner of the tosylhydrazone **26**. The palladium catalyzed reaction afforded **36** as a mixture of diastereomers in a 79% combined yield. The hydrogenation of this mixture with H_2_ over Pd/C afforded compound **37** at a 87% yield. The stereoselective dehydroxylation of **37** was carried out with Sn(OTf)_2_, giving the trisubstituted alkene **38** at a 90% yield. The optimization of the cyclization of **38** into **15** in terms of Si^4+^ reagent, solvent, and temperature led to the conclusion that at high temperatures, TMSOTf was the most efficient promotor in dichloromethane, giving the tetracyclic compound **15** at a 81% yield. With the deprotection of the aryl methyl ethers in **15** by treatment with BI_3_, the synthesis of (−)-pelorol (**1**) [19] was completed in nine steps (19.5% overall yield) from (+)-sclareolide (**5**). The optimization of this modular strategy allows the shortening of the number of reaction steps, considerably improving the global yield, and represents an excellent methodology for the synthesis of multiple natural compounds. However, the high cost of the transition metal used to catalyze the coupling reaction between **26** and **35** limits the use of this synthetic route.

### 2.5. Li’s Synthesis of (−)-Pelorol (***1***)

**Scheme 7:**
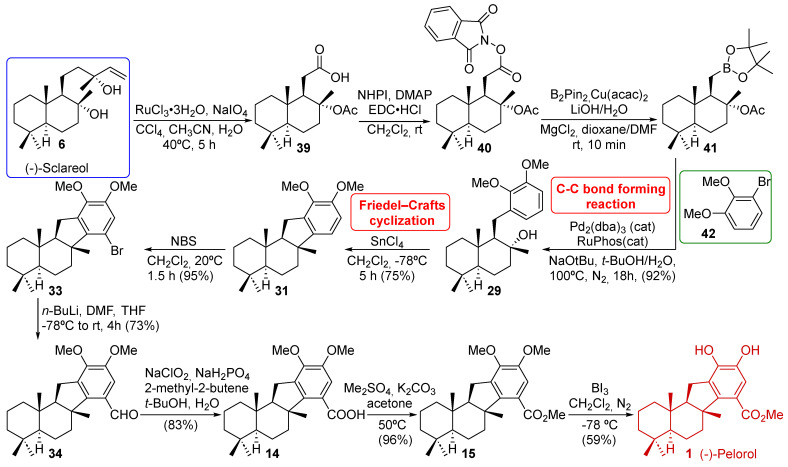
Li’s enantioselective synthesis of (−)-pelorol (**1**) from (−)-sclareol (**6**). NHPI = *N*-Hydroxyphthalimide; DMAP = 4-Dimethylaminopyridine; EDC.HCl = *N*-Ethyl-*N*′-(3-dimethylaminopropyl)carbodiimide hydrochloride; B_2_Pin_2_ = Bis (pinacolato)diboron; NBS = *N*-Bromosuccinimide; DMF = *N*,*N*-dimethyl formamide.

More recently, in 2024, Li and colleagues [20] reported an efficient synthesis of marine (−)-pelorol (**1**) from the inexpensive starting material (−)-sclareol (**6**). The key step was a Suzuki coupling of drimanyl Bpin **41** and arylbromide **42**. As shown in Scheme 7, drimanyl Bpin **41** can be generated in three steps from **6** through a degradative sequence of reactions, which includes a cascade oxidative process of (−)-sclareol (**6**) with RuCl_3_·3H_2_O to yield homodrimanic acid **39**. This acid can be converted to redox-active homodrimanyl-*N*-hydroxyphalimide ester **40** with *N*-hydroxyphthalimide (NHPT), and finally Cu-catalyzed decarboxylative borylation of **40** gives the desirable drimanyl Bpin **41**. This intermediate was prepared at a 27.5% overall yield, after the flash-column chromatography purification of the intermediates, or at a 25% overall yield, after the solvent partitioning/aqueous wash workup of the crude residues of the three steps. The C-C bond formation reaction by Suzuki coupling between drimanyl Bpin **41** and 1-bromo-2,3-dimethoxybenzene **42** gave the key intermediate **29** at a 92% yield. The aromatic derivative **42** was obtained from 3-bromocathecol at a 91% yield. Subsequent Friedel–Crafts-type cyclization of the key intermediate **29** with SnCl_4_ at −78 °C gave the tetracyclic indane-fused decalin drimane **31** at a 75% yield with the desired diastereoselectivity. The introduction of formaldehyde in the aromatic ring was achieved through a two-step bromination- and lithium–halogen exchange-promoted formylation. Thus, when compound **31** was treated with NBS, the bromine derivative **32** was formed (95% yield). The reaction of **32** with *n*-BuLi and *N*,*N*-dimethyl formamide gave aldehyde **34** at a 73% yield. The subsequent Pinnick oxidation [29] of **34** followed by esterification gave methylated pelorol **14** in two steps (80% overall yield). Finally, the demethylation of product **15**, previously reported by Andersen and colleagues [19], formed (−)-pelorol (**1**) with a 59% yield. The formal synthesis of (−)-pelorol (**1**) was completed in 10 steps, with a 5.6% overall yield.

The main drawback of this easy synthesis is the low yield of the three-step sequence required to prepare the drimanyl Bpin **41** from (−)-sclareol, even though it is scalable, and that the palladium catalyst employed in the coupling reaction between **41** and **42** has a high cost.

## 3. Future Perspectives

In light of the comprehensive review of the different synthesis of the marine (−)-pelorol (**1**) from the inexpensive and commercially available (+)-sclareolide (**5**) and (−)-sclareol (**6**) presented in Section 2, it is clear that the carbon skeleton of (−)-pelorol (**1**) can been successfully prepared through processes that include two key steps. The first one is the coupling of two partners (a drimanic derivative and an aromatic derivative) by different synthetic approaches that might include the addition of aryl-metallic reagents to a carbonyl group, the Suzuki coupling of the boron derivatives of the drimane portion, or the palladium-catalyzed tandem carbene migratory insertion of tosylhydrazone derivatives. The second key step is a Lewis acid-promoted C-C bond formation on the aromatic ring through a Friedel–Crafts reaction, with concomitant cyclization to form the C ring present in the tetracyclic indane-fused decalin drimane. The success of the first key step is due to the efforts made in the development of appropriate procedures for the preparation of the intermediates (**20**, **37**, **41**) that have been used in the divergent syntheses of this and other marine natural products. It is also worth mentioning that the introduction of the ester group in the aromatic ring before the cyclopentane ring formation by Friedel–Crafts cyclization (Scheme 6) can improve the overall yield of (−)-pelorol (**1**). The creation of the cyclopentane ring by the Friedel–Crafts reaction from an aromatic derivative with a deactivating substituent is possible thanks to the use of TMSOTf as an extremely effective additive.

**Scheme 8:**
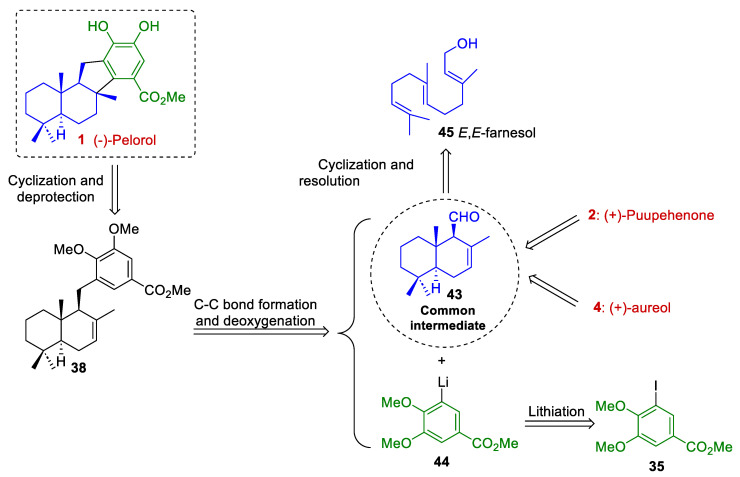
Retrosynthetic plan of (−)-pelorol (**1**), (+)-puupehenone (**2**) [38], and (+)-aureol (**4**) [36] from a common intermediate **43** [40,41].

However, despite all the advances made, we think that a general strategy for (−)-pelorol (**1**) and analogues in a concise and divergent way is still desirable. In this section, based on our personal experience in the synthesis of natural compounds [30,31,32,33,34,35,36,37] and on the antecedents discussed in Section 2, we propose a retrosynthetic analysis of **1** in six steps, using the common synthetic intermediate **43** as the starting material. This aldehyde **43** could allow the divergent synthesis of meroterpenoids such as (−)-pelorol (**1**), (+)-puupehenone (**2**) [38], and (+)-aureol (**4**) using procedures previously reported by us (Scheme 8) [36,37,38,39,40].

We propose that a convergent preparation of compound **1** is the best strategy for the obtention of this marine compound, since it can improve the overall yield and the efficiency of the multistep synthesis. Therefore, the key merosesquiterpene **38** could be prepared via addition of an aryllithium reagent **44** to the carbonyl group of **43**. The aldehyde **43** can be prepared in an optically pure form from *E*,*E*-farnesol **45** by a cationic cyclization reaction and the subsequent resolution of the racemic mixture via the chromatographic techniques of the diastereomeric camphanoate derivatives [41,42].

The aryllithium **44** can be synthesized through the lithiation of **35**. We consider the aromatic ring **44** to be the second partner in the coupling reaction with **43**, since it has been shown to be useful in the Friedel–Crafts cyclization, and it shortened the synthetic route to (−)-pelorol (**1**) (Scheme 6) [26]. The deoxygenation of the C15-OH group of the coupling product between **43** and **44** would yield the key intermediate **38**. The subsequent Friedel–Crafts cyclization and deprotection of the OMe groups would afford marine pelorol (**1**). Considering the retrosynthetic Scheme 8, it could be stated that the intermediate **43** would be a very valuable candidate in divergent synthesis. Its coupling with an appropriate aromatic partner would generate valuable intermediates for the synthesis of marine products with indane-fused decalin (e.g., (−)-pelorol (**1**)), the tetracyclic system with benzopyran-fused-naphthane (e.g., (+)-puupehenone (**2**) [38]), or the tetracyclic system with a substituted benzopyran moiety (e.g., (+)-aureol (**4**) [36,40].

The general retrosynthetic analysis reported in Scheme 8 represents a low-cost and powerful tool the synthesis of (−)-pelorol (**1**), which could be used to address its scarcely explored biological activities. This retrosynthetic analysis can also be easily used in the synthesis of an array of analogues of (−)-pelorol (**1**), simply by modifying the choice of the aromatic ring. Some derivatives with simple modifications of functional groups in their aromatic ring have been shown to be potential lead products for cancer therapy [21]. The quick and facile access to (−)-pelorol (**1**) and analogues through Scheme 8 would enable the establishment of a preliminary structure and activity relationships for further optimization.

## 4. Conclusions

A review of the synthesis of natural product (−)-pelorol (**1**) has been reported in terms of the synthetic procedure employed. Despite the successful synthesis of pelorol in a reasonable number of synthetic operations (9–12 steps; see Section 2) more practical access to this marine product is still desirable. Taking into consideration the best results from each strategy, we propose a concise synthesis of pelorol. The synthetic strategy is designed to require the minimum number of steps and is supported in mild conditions and operational easiness, which could be employed to address future biological activities.

## Data Availability

No new data were created.
